# Cosbin: cosine score-based iterative normalization of biologically diverse samples

**DOI:** 10.1093/bioadv/vbac076

**Published:** 2022-10-20

**Authors:** Chiung-Ting Wu, Minjie Shen, Dongping Du, Zuolin Cheng, Sarah J Parker, Yingzhou Lu, Jennifer E Van Eyk, Guoqiang Yu, Robert Clarke, David M Herrington, Yue Wang

**Affiliations:** Department of Electrical and Computer Engineering, Virginia Polytechnic Institute and State University, Arlington, VA 22203, USA; Department of Electrical and Computer Engineering, Virginia Polytechnic Institute and State University, Arlington, VA 22203, USA; Department of Electrical and Computer Engineering, Virginia Polytechnic Institute and State University, Arlington, VA 22203, USA; Department of Electrical and Computer Engineering, Virginia Polytechnic Institute and State University, Arlington, VA 22203, USA; Advanced Clinical Biosystems Research Institute, Cedars Sinai Medical Center, Los Angeles, CA 90048, USA; Department of Electrical and Computer Engineering, Virginia Polytechnic Institute and State University, Arlington, VA 22203, USA; Advanced Clinical Biosystems Research Institute, Cedars Sinai Medical Center, Los Angeles, CA 90048, USA; Department of Electrical and Computer Engineering, Virginia Polytechnic Institute and State University, Arlington, VA 22203, USA; The Hormel Institute, University of Minnesota, Austin, MN 55912, USA; Department of Internal Medicine, Wake Forest University, Winston-Salem, NC 27157, USA; Department of Electrical and Computer Engineering, Virginia Polytechnic Institute and State University, Arlington, VA 22203, USA

## Abstract

**Motivation:**

Data normalization is essential to ensure accurate inference and comparability of gene expression measures across samples or conditions. Ideally, gene expression data should be rescaled based on consistently expressed reference genes. However, to normalize biologically diverse samples, the most commonly used reference genes exhibit striking expression variability and size-factor or distribution-based normalization methods can be problematic when the amount of asymmetry in differential expression is significant.

**Results:**

We report an efficient and accurate data-driven method—Cosine score-based iterative normalization (Cosbin)—to normalize biologically diverse samples. Based on the Cosine scores of cross-condition expression patterns, the Cosbin pipeline iteratively eliminates asymmetric differentially expressed genes, identifies consistently expressed genes, and calculates sample-wise normalization factors. We demonstrate the superior performance and enhanced utility of Cosbin compared with six representative peer methods using both simulation and real multi-omics expression datasets. Implemented in open-source R scripts and specifically designed to address normalization bias due to significant asymmetry in differential expression across multiple conditions, the Cosbin tool complements rather than replaces the existing methods and will allow biologists to more accurately detect true molecular signals among diverse phenotypic groups.

**Availability and implementation:**

The R scripts of Cosbin pipeline are freely available at https://github.com/MinjieSh/Cosbin.

**Supplementary information:**

Supplementary data are available at *Bioinformatics Advances* online.

## 1 Introduction

High-throughput gene expression profiling provides the ability to study many genes in an organism under different biological conditions (Clarke *et al.*, 2008). Between-sample normalization is essential to ensure accurate inference and comparability of gene expression measurements across samples or conditions. Ideally, such normalization should be based on a subset of consistently expressed reference genes (CEGs) to correct for technical differences (non-biological) among samples (Evans *et al.*, 2018). Inaccurate normalization can significantly bias downstream analysis, potentially rendering the choice of test statistics for hypothesis testing (Zhao *et al.*, 2020).

**Fig. 1.**
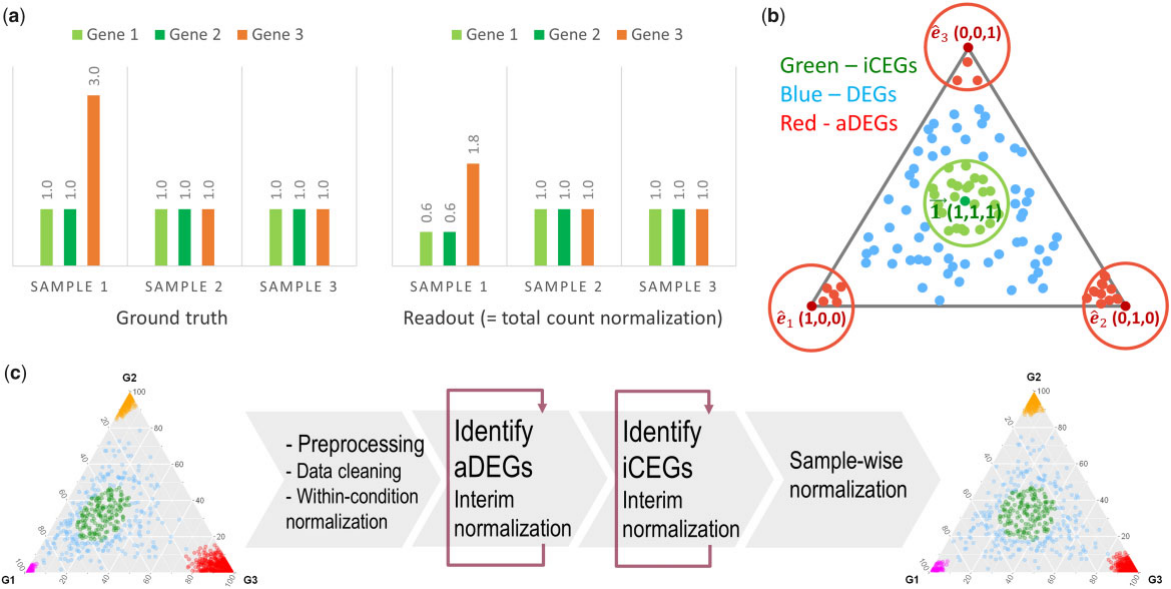
Cosbin concept and framework. (**a**) Toy illustration of asymmetric differential expression and resulting biased individual readouts: Genes 1 and 2 (green) are iCEGs, Gene 3 (orange) is aDEG, while common total readout count produces biased between-sample normalization where two iCEGs become differentially expressed and one aDEG becomes less differentially expressed. (**b**) Geometrical locations of significant aDEGs (red, corners), iCEGs (green, center) and general DEGs (blue, the rest), in the scatter simplex of data matrix. (**c**) Overall workflow of Cosbin algorithm with two major consecutive iterative loops

We and others have recognized that the direct application of size-factor-based or distribution-based normalization methods to biologically diverse samples can be problematic due to two major as-yet unresolved problems. First, the most commonly used reference genes are unreliable as internal controls for normalization because of their striking expression variability in both experimental and bioinformatic analyses (Jo *et al.*, 2019). Second, while it is conceptually logical to determine scaling factors or global transformations based on the global distribution of many expressed genes when the amount of asymmetry in the differential expression is high, this global adjustment (scaling or transformation) strategy may bias true differential expression toward the null (Fig. 1a) (Evans *et al.*, 2018; Hicks and Irizarry 2015; Johnson and Krishnan 2022). The data-driven assessment tool quantro was developed to guide the choice of an appropriate normalization method using global transformations (Hicks and Irizarry 2015). A few normalization-by-testing strategies have also been proposed to mitigate the impact of asymmetrically and differentially expressed genes (aDEGs) with limited success (Evans *et al.*, 2018; Sun *et al.*, 2013; Wang *et al.*, 2002).

To specifically address the impact of significant asymmetry in the differential expression on normalizing biologically diverse samples, we report here a Cosine score-based iterative normalization (Cosbin) method that eliminates aDEGs, identifies ideal CEGs (iCEGs) and calculates sample-wise normalization factors by equilibrating expression levels of iCEGs (Fig. 1). The Cosbin tool performs reference-free multiple normalizations across different biological conditions and is applicable to multi-omics data. As a novel normalization-by-testing strategy (Evans *et al.*, 2018), the Cosbin framework makes no assumption that the total expression is the same or that differential expression across differential experimental conditions is approximately symmetrical and thus complements rather than replaces existing methods (Evans *et al.*, 2018; Johnson and Krishnan 2022). One additional benefit of the Cosbin tool is the concurrent detection of marker genes (MGs) and iCEGs (Lu *et al.*, 2022) (Supplementary Information).

The Cosbin software is implemented in open-source R scripts (Fig. 1). We demonstrate the effectiveness of the Cosbin strategy, in comparison with six representative peer methods (three size-factor-based, two global-adjustment and one normalization-by-testing) (Evans *et al.*, 2018; Johnson and Krishnan 2022), using realistic mixture simulations and quantitative accuracy measures against ground truth. We also report biomedical case studies where Cosbin is used to normalize gene expression and proteomics data from biologically diverse samples (Herrington *et al.*, 2018; Parker *et al.*, 2020). The Cosbin tool will allow biologists to more accurately detect true molecular signals among diverse phenotypic groups.

## 2 Methods

### 2.1 Eliminating asymmetric DEG and interim normalization

Because a few highly expressed genes can account for a large share of total expression, overall differential expression is asymmetric across conditions (e.g. grouped or diverse samples) when the number or magnitude of up/down-regulated expressions in each condition is unequal (Fig. 1a, Supplementary Fig. S1) (Evans *et al.*, 2018). In the problem formulation, for simplicity, we focus our discussions on the basic yet challenging case of multiple grouped samples. However, our results hold for diverse individual samples.

Mathematically, an aDEG $i_{\text{aDEG},\;k}$ associated with group *k* is defined as a gene expressed significantly high in group *k* while universally low in any other groups (expressed in group *k* as exclusively as possible), approximately

(1) xkiaDEG, k≫0,xl≠kiaDEG, k≈0,

where, $x_{k}\left(i_{\text{aDEG},\;k}\right)$ and $x_{l\neq k}\left(i_{\text{aDEG},\;k}\right)$ are the averaged expressions of gene $i_{\text{aDEG},\;k}$ in groups *k* and l, respectively (Fig. 1). Accordingly, the cross-group expression pattern of an aDEG can be represented concisely by the Cartesian unit vectors ${\hat{e}}_{k}$, readily serving as the ground reference for scoring *de novo* aDEGs (Fig. 1b). Fundamental to the efficiency of Cosbin is the newly proposed score $\cos\left(x\left(i\right),\;{\hat{e}}_{k}\right)$ that measures directly the similarity between the cross-group expression pattern $x\left(i\right)=\left[x_{1}\left(i\right),\;x_{2}\left(i\right),\;\ldots ,\;x_{K}\left(i\right)\right]$ of gene *i* and the reference aDEG expression patterns of constituent groups in scatter space, where *K* is the number of constituent groups. Specifically, for gene *i* and group *k*, the aDEG score is given by

(2) $$t_{\text{aDEG}}(i)=\underset{1\leq k\leq K}{\mathrm{argmax}}\cos\left(x\left(i\right),\;{\hat{e}}_{k}\right)=\underset{1\leq k\leq K}{\mathrm{argmax}}\frac{x_{k}\left(i\right)}{\sqrt{\sum_{j=1}^{K}{\left[x_{j}(i)\right]}^{2}}},$$

where the ‘max’ operation in (2) is applied to address significantly asymmetric differential expressions among multiple groups. Because $x\left(i\right)$ is confined within the first quadrant where the central vector is the ‘all-ones’ vector $\vec{1}$, we have $1/\sqrt{K}<t_{\text{aDEG}}(i)<1$.

Impactful aDEGs with higher scores are sequentially identified and removed then interim normalization is performed by equilibrating expression levels for the remaining genes, and Cosbin iterates to the next round of aDEG identification and interim normalization (Wang *et al.*, 2002). Sequential elimination of impactful aDEGs should ease the asymmetry in differential expression across groups, reduce normalization bias and improve the efficiency of identifying the next aDEG. Iterations continue until aDEG identification or interim normalization converges at a stable point.

### 2.2 Identifying ideal CEGs and final normalization

We finalize normalization by identifying iCEGs and determining normalization factors across groups and individual samples. Mathematically, the cross-group expression pattern of an iCEG can be represented concisely by the ‘all-ones’ vector $\vec{1}$ (Evans *et al.*, 2018), serving as the ground reference for scoring CEGs (Fig. 1b). Accordingly, the iCEG score is given by

(3) $$t_{\text{iCEG}}(i)=\cos\left(x\left(i\right),\vec{1}\;\right)=\frac{\sum_{j=1}^{K}x_{j}\left(i\right)}{\sqrt{K\sum_{j=1}^{K}{\left[x_{j}\left(i\right)\right]}^{2}}},$$

where $1/\sqrt{K}<t_{\text{iCEG}}(i)<1$. Once iCEGs are identified, normalization is refined by equilibrating expression levels for iCEGs and the algorithm iterates to the next round of iCEG identification (Evans *et al.*, 2018; Wang *et al.*, 2002). The iterations between iCEG identification and re-normalization continue until iCEG identification or re-normalization converges at a stable point.

### 2.3 Cosbin workflow and software

The Cosbin workflow consists of six major analytic steps (Fig. 1c):

1. Initial normalization. An initial between-sample normalization is performed using total count (Evans *et al.*, 2018; Wang *et al.*, 2002).
2. Data cleaning. Genes whose norms $\left\|x\left(i\right)\right\|$ are lower or higher than pre-fixed thresholds are removed as noise or outliers.
3. aDEG score and removal. For each remaining gene, score ${\mathrm{argmax}}_{1\leq k\leq K}\;\cos\left(x\left(i\right),\;{\hat{e}}_{k}\right)$ is calculated and the top ranked aDEG is iteratively eliminated.
4. Interim normalization. The rescaling factor per condition/sample is determined by the total count of non-aDEGs and used to normalize the remaining genes.
5. iCEG score and selection. For each remaining gene, score $\cos\left(x\left(i\right),\;\vec{1}\right)$ is calculated and the top iCEGs are selected.
6. Final normalization. The rescaling factor per sample is determined by the total count of iCEGs and used to normalize all individual genes. Within the loop comprising Steps 5 and 6, a sequence of increasing score thresholds may be applied to stabilize normalization.

We implemented the Cosbin workflow in R script and performed a community-trial software testing (Supplementary Information). The R script is open-source at GitHub and is distributed under the MIT license. The Cosbin software tool is easy to use and applicable to multi-omics data. Importantly, a group label on each sample is required in the input, and the output file contains the scores for individual genes belonging to either aDEGs or iCEGs.

### 2.4 Performance index

We emphasize that the Cosbin strategy aims to address normalization bias due to the impact of significant aDEGs. In simulation studies, we proposed four quantitative criteria to comparatively evaluate the performance of Cosbin and peer methods against the ground truth embedded in realistic simulations (Supplementary Information). To determine the location correctness of iCEGs against ground truth, we used the (i) average Cosine score in degree and (ii) average mean-squared-error of log-fold-change (LFC-MSE), which measure the dislocation of iCEGs from the ground truth after normalization by Cosbin and peer methods (Supplementary Fig. S8) (Evans *et al.*, 2018). As these genes are iCEGs, if normalization is performed correctly then both the average Cosine score in degrees and LFC-MSE between samples of different conditions should be close to 0. For studying the impact of various normalization strategies on downstream differential analysis, we used (iii) the whole or partial receiver operating characteristics (ROC/pROC) curve and (iv) the area under ROC curve (AUC/pAUC) in order to assess the sensitivity and specificity of detecting general DEGs after normalization by Cosbin and peer methods (Evans *et al.*, 2018; Jo *et al.*, 2019). In our benchmarking studies, we proposed two quantitative criteria to evaluate the impact of different normalization methods on downstream marker gene (MG) detection among biologically diverse samples (Parker *et al.*, 2020), namely (v) P1 index and (vi) average deviation degree (ADD).

## 3 Results

We conducted three phased experiments to evaluate the performance of Cosbin and its R software tool, Specifically, we performed comparisons between Cosbin and six peer methods using realistic simulation data, assessment of the impact of Cosbin on detecting MGs among many groups using benchmark gene expression data, and a biomedical case study applying Cosbin to real proteomics data.

### 3.1 Comparative evaluation of Cosbin and peer methods using simulation data

We conducted extensive experiments to evaluate the performance of Cosbin and six peer methods using simulation data (Evans *et al.*, 2018) (Supplementary Information). As is widely recognized, the evaluation of biased normalization due to aDEGs in real data is very difficult in practice (Zhao *et al.*, 2020). Thus, the performance of methods addressing the impact of aDEGs must be quantitatively evaluated using simulation data embedded with ground truth (Sun *et al.*, 2013). The six peer methods in our comparison consisted of three size-factor-based approaches (i) Total Count (Evans *et al.*, 2018), (ii) Trimmed Mean of the M-values in edge R (TMM/edgeR) (Robinson and Oshlack 2010), (iii) Differential Expression analysis of RNA-Seq (DESeq2) (Love *et al.*, 2014); one normalization-by-testing approach, (iv) Differentially Expressed Gene Elimination Strategy (DEGES) (Sun *et al.*, 2013); and two global-adjustment or transformation approaches, (v) Cumulative-Sum Scaling (CSS) (Paulson *et al.*, 2013) and (vi) smooth quantile normalization (qsmooth) (Hicks *et al.*, 2018). DEGES is not a self-contained normalization method but an add-on preprocessing step to improve the TMM/edgeR method (Sun *et al.*, 2013).

Simulation data were generated either from a mixture of Dirichlet and truncated normal distributions (mixture simulation) or by using the simulateReadCounts function in Tag Count Comparison (TCC) R package (TCC simulation) (Sun *et al.*, 2013). Each simulated data matrix contained 1000 genes with 168 iCEGs (green, 16.8%), 232 general DEGs (blue, 23.2%) and 600 aDEGs (red/orange/pink, 60%), across 3 groups with 10 replicates per group. The iCEGs are determined by $\cos\left(x\left(i\right),\vec{1}\;\right)\geq 0.95$, and the percentages of upregulated aDEGs were 10%, 30% and 60% for each of the three groups, respectively. The aDEGs are generated from a truncated normal distribution centered at ${\hat{e}}_{k}$ in the mixture simulation (Supplementary Fig. S2) and are generated with a pairwise fold-change of 10 in TCC simulation (Supplementary Fig. S5) (Sun *et al.*, 2013).

**Fig. 2.**
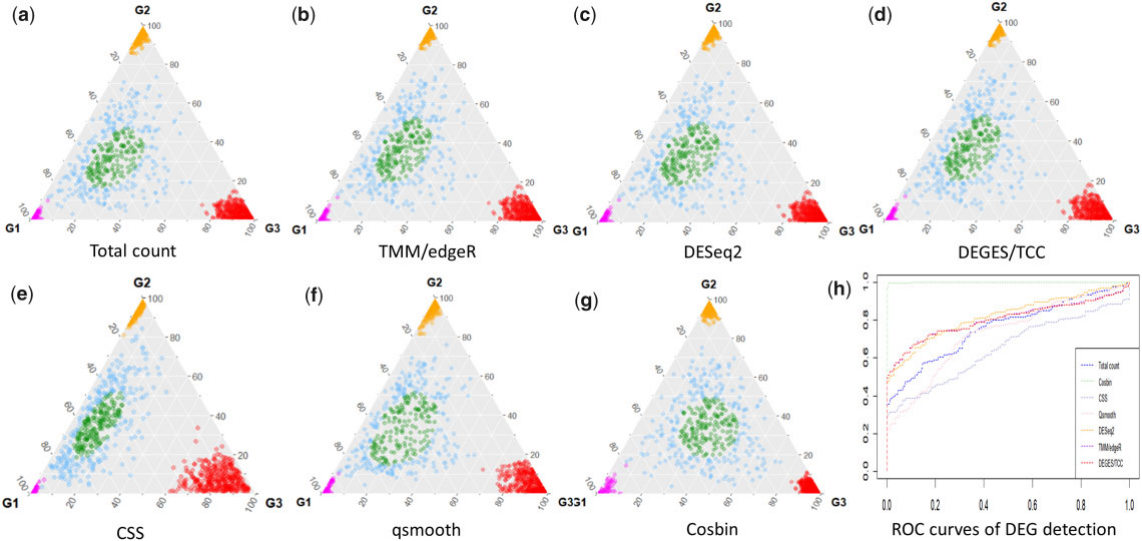
Comparative evaluation of Cosbin and six peer methods using mixture simulation data. (**a–g**) The location of iCEGs (green, # iCEGs = 168), significant aDEGs (red/orange/pink, # aDEGs = 600), and general DEGs (blue, # DEGs = 232), restored by Cosbin and six peer methods after normalization. (**h**) The ROC curves of detecting general DEGs against iCEGs by Cosbin and six peer methods

The average deviation-in-degree of $\cos\left(x\left(i\right),\vec{1}\;\right)$ values were used to evaluate the accuracy of iCEG restoration and discriminative accuracy was evaluated using both ROC/pROC curves and AUC/pAUC values to assess ability to distinguish between general DEGs and iCEGs (e.g. blue versus green) after normalization by Cosbin and six alternative and previously published (peer) methods (Supplementary Information). The experimental results are summarized in Table 1, Supplementary Table S1 and S2, Figure 2, and Supplementary Figure S4. In both mixture and TCC simulation studies, Cosbin consistently outperforms all six peer methods in terms of lower average dislocation of iCEGs (Table 1, Supplementary Tables S1 and S2) and higher AUC/pAUC of detecting DEGs (Table 1, Fig. 2h, Supplementary Fig. S2). Note that the relative performances of the six peer methods assessed in our experimental comparisons are consistent with previous reports in similar comparison studies (Evans *et al.*, 2018; Johnson and Krishnan 2022; Sun *et al.*, 2013). Notably, in terms of addressing the significant asymmetry in differential expression across multiple conditions, three popular size-factor-based methods (Total count, TMM/edgeR and DESeq2) perform relatively well, while two popular distribution-based methods (CSS and qsmooth) do not perform well. The poor performance of CSS is expected because CSS was designed specifically to normalize ‘sparse’ expression profiles (marker-gene survey data) (Paulson *et al.*, 2013) (Supplementary Information).

**Table 1.** Average dislocation of iCEGs (no. of iCEGs = 168) in degree and AUC of detecting general DEGs (no. of DEGs = 232) after normalization by Cosbin and six peer methods, summarized from mixture simulation studies

Methods	Average dislocation of iCEGs in degree (mixture simulation)	AUC of detecting general DEGs (mixture simulation)
Total count	18.29°	0.75
TMM/edgeR	17.46°	0.81
DESeq2	15.28°	0.82
DEGES/TCC	17.63°	0.80
CSS	28.81°	0.65
qsmooth	18.91°	0.73
Cosbin	1.42°	0.99

*Note*: Note that the maximum possible iCEG dislocation in this case is about 55°.

While DEGES performed comparatively well in the TCC simulation study (Supplementary Fig. S2, Supplementary Table S2), it performed poorly in the mixture simulation study (Fig. 2d and h, Table 1, Supplementary Table S1). This outcome was expected because TCC simulation may not represent a typical case of biologically diverse samples, see Supplementary Figure S1 (more blue-DEGs in mixture simulation) versus Supplementary Figure S5 (fewer blue-DEGs in TCC simulation). However, TCC simulation may not represent a mature simulation tool for analyzing multiple conditions, as acknowledged previously by its authors (Sun *et al.*, 2013). Additional experimental results and discussion are presented in Supplementary Information (Supplementary Figs S3, S6 and S7, Supplementary Tables S1 and S2).

We next assessed how the performance of Cosbin changes as the number of iCEGs changes, such as when the iCEG subset is very small in either total number or as a proportion. To this end, we have conducted additional experiments using different numbers (50, 100 and 150) and proportions (5%, 10% and 15%) of iCEGs identified and used for determining normalization factors in the Cosbin pipeline applied to our simulation studies. The experimental results are summarized mainly in Figure S4, and additionally in Figure 2 and Table 1 (# iCEGs = 168, the fixed number of simulated iCEGs). These results show that the performance of Cosbin remains robust even when using a small number of iCEGs, as was quantitatively measured by both the accuracy of iCEG restoration (average dislocation) and AUC values of DEG detection. To evaluate how the performance of global adjustment methods changes with different amounts of asymmetry in differential expression, we conducted simulation studies with varying level of aDEG imbalance. The experimental results on CSS and qsmooth are summarized in Supplementary Figures S11 and S12.

### 3.2 Assessment of Cosbin impact on marker gene quality using benchmark dataset

We assessed the impact of between-sample normalization by Cosbin and two peer methods (Total Count and DEGES/TCC) on MG quality using a benchmark gene expression dataset (GSE28490 comprised of *K *=* *5 groups). Total Count represents the baseline method and DEGES/TCC represents the most competitive peer method (Evans *et al.*, 2018; Zhao *et al.*, 2020). The definition of MG and related discussion are given in Supplementary Information. The quality of MGs is evaluated quantitatively by:

(4) $$P_{1}=\frac{1}{M(K-1)}\sum_{i=1}^{M}\left(\sum_{j=1}^{K}\frac{x_{j}\left(i_{\text{MG}}\right)}{{\text{max}}_{k}x_{k}\left(i_{\text{MG}}\right)}-1\right)$$

where, M is the number of MGs, and $0\leq P_{1}\leq 1$. By the definition of MG (Parker *et al.*, 2020), an ideal MG expression matrix corresponds to a row-permutation matrix with its minimum zero value attained using the $P_{1}$ index. The larger the $P_{1}$ value, the poorer the quality of MGs.

**Fig. 3.**
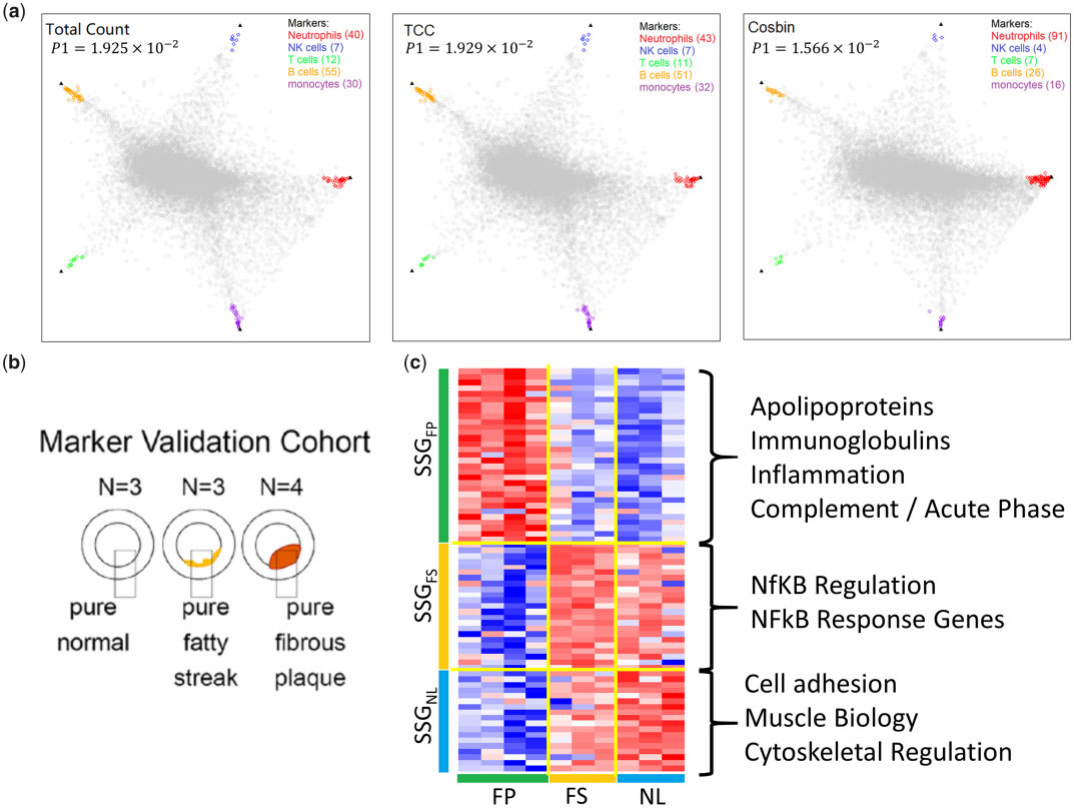
Case study of Cosbin impact on the quality of MGs and SSGs using gene expression and proteomics data. (**a**) Scatter simplex of GSE28490 superimposed by the top 144 MGs (color-coded) detected after normalization by total count, DEGES/TCC and Cosbin, respectively; where black triangle indicates the ideal MG references. (**b**) Anatomic description of the specimens. (**c**) Heatmap of top 72 SSGs detected after normalization by Cosbin on vascular proteomics data

The top 144 MGs were detected after normalization by total count, TCC and Cosbin; the geometric proximity of these MGs to the vertices of the scatter simplex is shown in Figure 3a (color-coded). Results show that Cosbin outperforms the Total Count and DEGES/TCC in terms of higher MG quality. Notably, Cosbin achieves a lower $P_{1,\;\mathrm{Cosbin}}=1.566\times 10^{-2}$ when compared with $P_{1,\;\mathrm{TCC}}=1.929\times 10^{-2}$ and $P_{1,\;\mathrm{total}-\mathrm{count}}=1.925\times 10^{-2}$. These improvements by Cosbin correspond to a relative reduction of 18.8% over TCC and 18.6% over total count in terms of $P_{1}$ index. We further calculated the ADD of the top 144 MGs in relation to the ideal reference, which is summarized in Supplementary Table S4. Again, Cosbin achieves a much lower ${\mathrm{ADD}}_{1,\;\mathrm{Cosbin}}=2.4210$ when compared with ${\mathrm{ADD}}_{1,\;\mathrm{TCC}}=2.7330$ and ${\mathrm{ADD}}_{1,\;\mathrm{total}-\mathrm{count}}=2.6980$. These improvements by Cosbin correspond to a relative reduction of 11.4% over TCC and 10.3% over total count in terms of the ADD.

This benchmarking study also indirectly shows consistent relative performances of these methods, as was also observed in the mixture simulation study, and that DEGES/TCC appears to be less effective for normalizing biologically diverse samples. Additional experimental results and discussion are presented in Supplementary Information (Supplementary Fig. S9, Supplementary Table S4).

### 3.3 Case study of Cosbin on subtype signature genes using vascular proteomics data

We further demonstrated the utility of Cosbin on normalizing experimentally acquired proteomics data from biologically diverse samples for the purpose of detecting subtype signature genes (SSGs). The dataset was obtained from a cohort of ‘pure’ fibrous plaque (FP, *n* = 4), fatty streak (FS, *n* = 3), and normal (*n* = 3) vascular specimens (Fig. 3b) (Parker *et al.*, 2020). After normalization over 824 proteins by Cosbin, 31 SSGFP, 23 SSGFS and 18 SSGNL, are detected. The corresponding heatmap is shown in Figure 3c, the scatter simplex with color-coded SSG is given in Supplementary Figure S10, and the protein names are listed in Supplementary Table S3.

Closer interrogation of the functions associated with the 72 SSG linked to each subtype underscores the biological validity of this analysis. Excitingly, this analysis allowed us to find some very interesting hits in FS with a convincing link to Nuclear Factor of kappa B (NFκB), an established mediator of early atherogenesis. The 14 of 23 SSGFS could be associated with NFκB signaling, including regulators of NFκB activation such as MCAM, CAST, KTN1 and NDRG1; coactivators of NFκB transcriptional activity DHX9 and DDX1; and numerous known NFκB response genes UGDH, CPNE3, ACAN, TMOD1, GNB2, CYBRD1, SND1 and TUBB6. The NFκB pathway is implicated in early atherogenesis as a mediator of proinflammatory signaling due to the retention of oxidized lipid species. Of particular note, MCAM/CD146 is known to be a major NFκB coactivator and has been identified as a key molecule in the promotion and retention of foam cells, which are the definitive feature of the FS tissue samples. The FP stage of the disease has been well characterized by our group (Herrington *et al.*, 2018; Parker *et al.*, 2020), to be upregulated in expression of proteins associated with the acute phase (e.g. C7, A2M and SAA4), immune (e.g. immunoglobulin genes), and apolipoprotein accumulation (e.g. APOB, APOD, APOE, APOA1/2 and APOC3). The SSGNL have functions related to cell adhesion (LIMS1, ITGA7, SPARCL1, SPON1 and TLN2), muscle cell biology (KRT8, MYL9 and SLMAP) and cytoskeletal organization (PDLIM1, SYNM, VASP and NEXN).

Overall, the SSGs identified here reflect the established biological distinctions between a quiescent contractile healthy normal artery, the early stages of inflammatory response observed in FSs and the late-stage inflammatory and immunogenic biology of later-stage FPs. Furthermore, these data support prior contentions that early intervention at the level of FS accumulation by the use of NFκB modulating drugs may be a viable strategy for slowing atherosclerotic progression.

## 4 Discussion

The Cosbin R package provides an efficient and accurate tool for normalizing biologically diverse samples, specifically addressing the challenge of bias due to significant asymmetry in differential expression across multiple conditions (Evans *et al.*, 2018; Johnson and Krishnan 2022). Our study demonstrated that the elimination of significantly aDEGs is essential for obtaining good normalized data across biologically diverse samples. The proposed cosine-based test scores $\cos\left(s\left(i\right),\;{\hat{e}}_{k}\right)$ or $\cos\left(x\left(i\right),\vec{1}\;\right)$ matched exactly the definition of significantly aDEGs or iCEGs. The experimental results show that Cosbin outperforms existing peer methods, particularly for addressing biased normalization due to significant asymmetry in differential expressions. While the case studies here involve only transcripts and proteins, the Cosbin method and software tool are readily applicable to other omics data types.

Biologically diverse samples may be defined by two subtly different factors: a large portion of symmetric differential expression or a large portion of asymmetry in differential expressions (Evans *et al.*, 2018). Global adjustment methods have been developed specifically for use when there are many symmetrically and differentially expressed genes. We thus recommend that users apply the quantro tool in a first attempt prior to Cosbin in order to readily assess whether some existing global adjustment methods designed for normalizing biologically diverse samples (containing many general DEGs but not aDEGs) are readily applicable (Hicks and Irizarry 2015; Hicks *et al.*, 2018).

In relation to previous work, our goal of eliminating significant DEGs from determining normalization factors has also been addressed in prior work (Sun *et al.*, 2013; Wang *et al.*, 2002). For example, pairwise fold change has been used to detect and eliminate DEGs, and an iterative total count on remaining genes is calculated to normalize samples between two groups (Wang *et al.*, 2002). Similarly, in DEGES, conventional statistical testing is first applied to detect and eliminate DEGs; various size-factor-based methods are then used to perform normalization (Sun *et al.*, 2013). The unique property of Cosbin is that two cosine scores are specifically designed and used, one to detect and eliminate aDEGs and another to detect iCEGs, across multiple conditions and the final sample-wise normalization factors are determined only by the total count of iCEGs. Furthermore, Cosbin is readily applicable to datasets containing missing values, with the assumption that a sufficient number of iCEGs contain no missing value, where normalization factors are calculated by Cosbin using a complete data matrix and then used to normalize the full data matrix in both interim and final normalizations.

Potential limitations associated with the assumptions made by the current Cosbin design include that no excessive global shift in expression occurs (Evans *et al.*, 2018), the distribution of remaining general DEGs is approximately ‘symmetric’, a sufficient number of iCEGs exist and technical effects impact iCEGs and general DEGs alike across all biologically diverse samples. As a follow-up step, batch effect adjustment may be performed when needed (Clarke *et al.*, 2008). For normalizing samples across multiple conditions, a two-stage phased normalization strategy is recommended in which within-condition samples (biologically similar) are firstly normalized using Total Count and then between-condition samples (biologically diverse) are then normalized using Cosbin (Zhao *et al.*, 2020). For evaluating normalization performance, while PCR data are often used as a ‘gold standard’ to determine ‘true’ differential expression, we note that the practice of treating PCR as the ‘ground truth’ may not always be justified: there has been concern over possible errors in PCR data (Evans *et al.*, 2018).

## Funding

This work was supported by the National Institutes of Health [HL111362-05A1, HL133932 and NS115658-01]; and the Department of Defence [W81XWH-18-1-0723 (BC171885P1)].

*Conflict of Interest*: none declared.

## Supplementary Material

vbac076_Supplementary_Data

## Data Availability

Benchmark dataset used in this paper can be downloaded from Gene Expression Omnibus (https://www.ncbi.nlm.nih.gov/geo/) under the accession number: GSE28490.
