# Supplementary material for: Cosbin: cosine score-based iterative normalization of biologically diverse samples
Source: Bioinform Adv. 2022 Oct 20;2(1):vbac076. doi: 10.1093/bioadv/vbac076 (PMC9614059; doi:10.1093/bioadv/vbac076)
Supplement: vbac076_Supplementary_Data [file vbac076_supplementary_data.docx]

Supplementary Information

**Cosbin: Cosine score based iterative normalization of biologically diverse samples**

Chiung-Ting Wu, Minjie Shen, Dongping Du, Zuolin Cheng, Sarah J. Parker, Yingzhou Lu, Jennifer E. Van Eyk, Guoqiang Yu, Robert Clarke, David M. Herrington, Yue Wang

**Contents**

[Methods 2](#_Toc114480564)

[Results 4](#_Toc114480565)

[Discussion 8](#_Toc114480566)

[R Scripts 10](#_Toc114480567)

[Supplementary Tables 12](#_Toc114480568)

[Supplementary Figures 17](#_Toc114480569)

[References 24](#_Toc114480570)

## Methods

**Motivations**

The observations in quantro studies explicitly support the motivations and intentions of Cosbin (Hicks and Irizarry, 2015): (i) "When comparing the gene expression of two tissues, we found striking global differences in the distributions between brain and liver tissues", (ii) "if a substantially higher percentage of genes are expected to be expressed in only one group of samples it may not be appropriate to use global adjustment methods", (iii) "if the total RNA per cell is not the same across the samples, we suggest using control genes or spike-in controls".

**Performance index**

We propose four quantitative criteria to evaluate Cosbin performance based on the ground truth imbedded in the simulations or golden standard in real data. First, with respect to the correctness of ideal CEGs (iCEGs), we use the average Cosine score and/or corresponding degree to measure the ‘reduced’ dislocation of iCEGs after normalization by Cosbin in comparison with peer methods. Second, to measure how well the normalization methods equilibrate the total read counts of iCEGs, we calculate the pairwise (e.g., for three groups G1, G2, and G3, pairwise comparison means G1 vs. G2, G1 vs. G3, G2 vs. G3) average log-fold-change (LFC) mean squared error (MSE) (compare LFC to 0) for iCEGs across groups, then average these pairwise LFC MSE (Figure S8) (Evans, et al., 2018). Third, to study the effects of normalization on downstream analysis, we use the whole or partial Receiver Operating Characteristics (ROC) curve and the corresponding Area Under Curve (AUC) value to assess the sensitivity and specificity of detecting general DEGs after normalization by Cosbin in comparison with peer methods (Evans, et al., 2018; Jo, et al., 2019).

**Six representative peer methods (divided into three categories)**

- **Total count** (normalization by size-factor): This method uses the total read counts summed from all genes in a sample (library size) as the normalization factor to normalize that sample (Evans, et al., 2018).
- **DESeq2** (normalization by size-factor): DESeq2 uses the geometric mean of all samples as a pseudo-reference sample; then for each gene, calculates the ratio of each sample to the reference sample; lastly, rescale each sample using the median of the gene ratios associated with that sample (Anders and Huber, 2010).
- **TMM/edgeR** (normalization by size-factor): Trimmed Mean of the M-values (TMM) selects a sample as the reference sample; for each gene, calculates the fold change of each sample to the reference sample; “trimming” the genes (DEGs in relation to the reference sample); lastly rescale each sample using the trimmed mean of the fold change (Robinson and Oshlack, 2010). The edgeR is the R package implements TMM.
- **DEGES** (normalization by testing): Differentially Expressed Genes Elimination Strategy (DEGES) normalizes tag count data by first eliminating the DEGs then performs normalization. The package incorporates multi-step normalization methods, whose strategy is to remove potential DEGs before performing the data normalization. Tag Count Comparison (TCC) is the R package implements DEGES (Kadota, et al., 2012; Sun, et al., 2013).
- **CSS** (normalization by distribution): Cumulative-Sum-Scaling (CSS) aims specifically to normalize biologically diverse samples with ‘highly sparse’ profiles (e.g. marker-gene survey data), by distribution-guided scaling with the cumulative sum of counts up to a percentile or *k*th quantile (e.g. adaptive extension of scale counts by the 75th percentile of nonzero count distribution) (Paulson, et al., 2013).
- **qsmooth** (normalization by distribution): smooth quantile normalization (qsmooth) is a generalization of quantile normalization (global transformation), aimed at removing technical variance while retaining biological variance between grouped samples, by globally matching each of the quantiles corresponding to the 'consistent quantiles' while zero-weighting out the differential quantiles (Hicks, et al., 2018). qsmooth was designed for use when there are not known reference genes and a lot of differentially expressed genes.

**Design of Simulation data and Experiments**

To validate the efficacy of Cosbin, we evaluated the performance of Cosbin and other six representative normalization methods (Total Count, DESeq2, TMM/edgeR, DEGES/TCC, CSS, qsmooth) via two simulation studies (mixture simulation and/or TCC simulation) (see the illustrative scatter simplex plots in Figures S1 and S5). Same parameter setting is used in both simulations. The simulations contain three groups (G1 vs. G2 vs. G3), 1000 genes, and 30 samples (10 replicates for each group). Of the 1000 genes, 60% (= 600) are aDEGs. The numbers of aDEGs between groups are set to be asymmetric, e.g., 6% (= 60) of the aDEGs are up-regulated in G1, 18% (= 180) are up-regulated in G2, and 36% (= 360) are up-regulated in G3. The genes with $t_{\text{iCEG}}\left( i \right)\geq0.95$are considered iCEGs (=168, 16.8%), and $t_{\text{iCEG}}\left( i \right)<0.95$are considered general DEGs (=232, 23.2%). Because the R packages edgeR, DESeq2, DEGES, CSS, qsmooth, all can be extended to analyze multi-group samples, the full dataset (with replicates) is fed into each of the normalization methods. Super samples (the average of all the replicates per group) are used for evaluation purpose. The scatter simplex of a toy dataset is given in Figure S1a.

All the R scripts used to conduct and produced these experimental results are provided (https://github.com/MinjieSh/Cosbin):

1. Cosbin_functions.R;
2. Cosbin_functions_evaluation.R;
3. Cosbin_functions_visualization.R;
4. Comparison_Experiment.R.

**Relationship to the concept of marker or signature genes**

Marker Gene (MG) or Signature Gene (SG) refers to the genes whose expression is highly and exclusively enriched in a particular cell population in the tissue of interest, but not in any others (Hunt, et al., 2019; Kuhn, et al., 2012; Kuhn, et al., 2011; Lu, et al., 2022; Newman, et al., 2015; Newman, et al., 2019; Wang, et al., 2021; Zhong, et al., 2013). MGs or SGs may be considered as a subset of significant aDEGs.

## Results

**Comparative evaluation using mixture simulations**

The significantly asymmetric DEGs are drawn from nonnegative normal distributions centered at ${\hat{\boldsymbol{e}}}_{k}$, and the remaining genes are drawn from a Dirichlet distribution with parameter $\alpha=(3, 3, 3)$. The scatter simplex of the ground truth and original readout is given in Figure S1b.

The experimental results are summarized in Figure 2, Table 1, Figure S2, Figure S3, Figure S6, Figure S7. Cosbin consistently outperforms all the six peer methods across different evaluation criteria. Notably, Cosbin successfully moved the iCEGs back to the center, and hence has the smallest iCEG dislocation in relation to the ground truth. As demonstrated by the comprehensive comparative evaluation studies reported by Evans et al., “normalization by testing can tolerate a larger difference in number of up- and down-regulated genes for higher proportions of DEG than can normalization by distribution” (Evans, et al., 2018). In contrast yet expectedly, all peer methods produced higher iCEG dislocations because the simulation data set was designed to have asymmetry in differential expression across the three groups, where the specific assumptions made by the peer methods are largely violated resulting in obvious and significant iCEG dislocations in the scatter simplex. The three popular size-factor-based methods, i.e., DESeq2, TMM/edgeR, and DEGES, performed reasonably well with respect to iCEG dislocation measures. Indeed, if amounts of asymmetry in differential expression is substantially high, it may not be appropriate to use global adjustment method (Hicks and Irizarry, 2015).

The average of LFC MSE measures the ability of equilibrating the ground truth iCEGs across groups after normalization. As shown Table S1 and Table S2, Cosbin has the lowest error. This shows that after Cosbin normalization, the ground truth iCEGs was corrected to be consistently expressed across groups as expected. In terms of the average LFC-MSE, the performances of TMM/edgeR and DEGES/TCC are even worse than total count.

We also compared the detection of general DEG (neither iCEGs nor aDEGs subject to the varying cosine score threshold) after normalization with the ground truth DEGs to create ROC curves for each method. According to the ROC curves, Cosbin has the highest top-left ROC partial curve and AUC, significantly outperform all peer methods (Figure 2h, Table 1). It should be pointed out that concerning the detection of general DEG this study aims to illustrate the impact of significant asymmetry in differential expression (biased normalization) on detecting relatively subtle DEGs (blue versus green) - a challenging case.

**Evaluation on the robustness of Cosbin performance**

To assess how the performance of Cosbin changes as the number of iCEGs changes via simulation studies, i.e., what happens when iCEG subset is very small (either in total number, or as a proportion), we have conducted additional experiments using varying number of iCEGs (50, 100, 150; or 5%, 10% and 15%; the actual number of iCEGs identified and used for determining normalization factors) in the Cosbin pipeline. The experimental results are summarized mainly in Figure S4, and additionally in Figure 2 and Table 1 (# iCEGs = 168, the fixed number of simulated iCEGs). These results show that the performance of Cosbin remains robust even when using a small number of iCEGs, quantitatively measured by both the accuracy of iCEG restoration (average dislocation) and AUC values of DEG detection.

**Comparative evaluation using TCC simulations**

The TCC simulation data were generated using R function *simulateReadCounts* in the TCC R package (Sun, et al., 2013). TCC produces the empirical distribution of read counts from *Arabidopsis* gene expression data, where the differential expression levels across three groups are 15-fold default in this study (Sun, et al., 2013). The scatter simplex of the ground truth and original readout is given in Figure S2 and Figure S5. It is important to note that TCC simulation does not represent a mature tool for multiple conditions, as acknowledged by the authors (Sun, et al., 2013).

The relative performance of Cosbin and four peer methods on TCC simulation data is similar to that on mixture simulation data except for DEGES/TCC. The experimental results are summarized in Figure S2, Figure S6, Figure S7, and Table S2. Again, Cosbin consistently outperforms all four peer methods across different evaluation criteria, followed by DEGES/TCC, TMM/edgeR, and DESeq2, and Total count has the worst performance in most cases. It is worth mentioning that DEGES yields the second-best performance should be expected because the simulation data were generated by the DEGES/TCC package. Notably, Cosbin successfully moved the iCEGs back to the center, and hence has the smallest iCEG dislocation compared with the ground truth. Total count has the largest iCEG dislocation. Under this simulation setting, DEGES/TCC, TMM/edgeR, and DESeq2 performed much better than Total count.

As shown in Table S2, Cosbin has the lowest LFC-MSE, followed by DEGES/TCC, TMM/edgeR, DESeq2, and Total count. While DEGES/TCC performs comparatively well as expected, both popular TMM/edgeR and DESeq2 methods produces significantly biased normalization.

We also compared the detection of all DEGs (general DEGs and aDEGs versus iCEGs, subject to varying cosine score threshold) after normalization against the ground truth DEGs to create ROC curves for each method. According to the ROC curves, Cosbin has the most top-left ROC partial curve, and the peer methods perform well while not as good as Cosbin (Figure S7). Again, concerning DEG detection this study represents an easy case because aDEGs represent very strong signals and were included in the ROC calculation.

**Assessment of Cosbin impact on marker gene quality**

On benchmark gene expression dataset (GSE28490 consisted of *K*=5 groups), the geometric proximity of the 288 MGs, detected after normalization by total count, TCC, and Cosbin, to the vertices of the scatter simplex is shown in Figure S9 (color-coded). Quantitatively, Cosbin achieves a much lower $P_{1,\mathrm{Cosbin}}=2.749\times{10}^{-2}$, as compared with $P_{1,\mathrm{TCC}}=3.313\times{10}^{-2}$ and $P_{1,total-count}=3.332\times{10}^{-2}$. These improvements by Cosbin correspond to a relative reduction of 17% over TCC and 17.5% over total count in terms of $P_{1}$ index. We further calculated the average deviation degree of the top 288 MGs in relation to the ideal reference, summarized in Table S4. Again, Cosbin achieves a much lower ${ADD}_{1,\mathrm{Cosbin}}=4.22⁰$, as compared with ${ADD}_{1,\mathrm{TCC}}=4.88⁰$ and ${ADD}_{1,total-count}=4.90⁰$. These improvements by Cosbin correspond to a relative reduction of 13.56% over TCC and 13.87% over total count in terms of the average deviation degree.

**Case study of Cosbin on vascular proteomics data**

Closer interrogation of the functions associated with the SSGs associated with each sample type underscores the biological validity of this approach. The SSGs identified from NL samples have functions related to cell-adhesion (LIMS1, ITGA7, SPARCL1, SPON1, TLN2), muscle cell biology (KRT8, MYL9, SLMAP), and cytoskeletal organization (PDLIM1, SYNM, VASP, NEXN) according to their annotated functions in the Uniprot database. In the FS samples, the majority SSGs (14 of 23) could be associated with Nuclear Factor of kappa B (NFkB) signaling. The list includes regulators of NFkB activation such as MCAM (PMID: 28084332), CAST (PMID: 8641452), KTN1 (PMID: 34219129), and NDRG1(PMID: 30561520), coactivators of NFkB transcriptional activity DHX9 (PMID: 27034008) and DDX1 (PMID: 19058135), and numerous known NFkB response genes (UGDH, CPNE3, ACAN, TMOD1, GNB2, CYBRD1, SND1, TUBB6)( PMID: 27374120, PMID: 23160072). The NFkB pathway is implicated in early atherogenesis as a mediator of proinflammatory signaling due to retention of oxidized lipid species (PMID: 21636112, PMID: 15731497). Of particular note, the SSG MCAM, otherwise known as CD146, is known to be a major NFkB coactivator and it has been identified as a key molecule in the promotion and retention of foam cells, which are the definitive feature of the FS tissue in this group (PMID: 28084332). The FP stage of the disease has been well characterized by our group (Parker, Chen, et al, 2020; Herrington et al, 2018) and others (PMID: 29129081, to be upregulated in expression of proteins associated with the acute phase (e.g., C7, A2M, SAA4), immune (e.g., immunoglobulin genes), and apolipoprotein accumulation (e.g, APOB, APOD, APOE, APOA1/2, APOC3). Overall, the SSGs identified here reflect the well-known biological distinctions between a quiescent contractile healthy normal artery, the early stages of inflammatory response observed in fatty streaks, and the late stage inflammatory and immunogenic biology of later stage fibrous plaques. Furthermore, these data support prior contentions that early intervention at the level of FS accumulation by use of NFkB modulating drugs may be a viable strategy for slowing atherosclerotic progression (PMID: 21636112).

## Discussion

The fundamental assumptions underlying DESeq2 and TMM/edgeR include: (1) Most genes are not DEG; (2) DEG and non-DEG genes behave similarly; and (3) Balanced expression changes, that is, the number and magnitude of up- and down-regulated genes are comparable. Normalization methods would perform poorly when the assumptions above are violated (Evans, et al., 2018; Zhao, et al., 2020).

Notably, while the relative performance of peer methods in the mixture simulation study is similar to the comparisons reported by Evan et al. (Evans, et al., 2018) (e.g., no clear winner, total count performs well) and is more 'expected' in the TCC simulation (e.g., total count is significantly biased, DEGES/TCC is the winner), DEGES/TCC performance differs remarkedly between the mixture and TCC simulation studies. Although the true reasons are not known yet, by a closer look at the relevant scatter simplex, Table 1, and P1 values in Figure 3a, TCC simulation may not represent a realistic case (e.g., empirical distribution is more on 'replicates', fewer 'blue' DEGs - non-iCEG/non-iDEG) and actually all methods perform reasonably well. Furthermore, in the benchmark study (GSE28490, DEGES/TCC appears to be less effective for addressing symmetric differential expression, producing P1 value even slightly higher than that with total count.

One possible reason may be related to 'blue-DEG abundance/asymmetry'. When there are many blue-DEGs, determining normalization factor using all remaining genes in DEGES/TCC may be suboptimal. Thus, the additional step of identifying iCEGs in Cosbin may be beneficial (e.g. mixture simulation), yet less useful when there are fewer blue-DEGs (e.g. TCC simulation). Moreover, the benchmark data (GSE28490) represents biologically diverse groups including many blue-DEGs, and we see that DEGES/TCC has a P1 value comparable to that of total count and higher than that of Cosbin.

## R Scripts

The R scripts are available at https://github.com/MinjieSh/Cosbin

Below is the Cosbin result on a simple toy dataset and the workflow. This toy dataset example contains different numbers of replicates in each group, specifically, 1 sample in group 1, 2 samples in group 2, and 3 samples in group 3. Among the 4 genes, there are 2 CEGs, 1 significantly asymmetric DEG, and 1 general DEG.

More suggestions on parameter setting can be found in the package vignette.


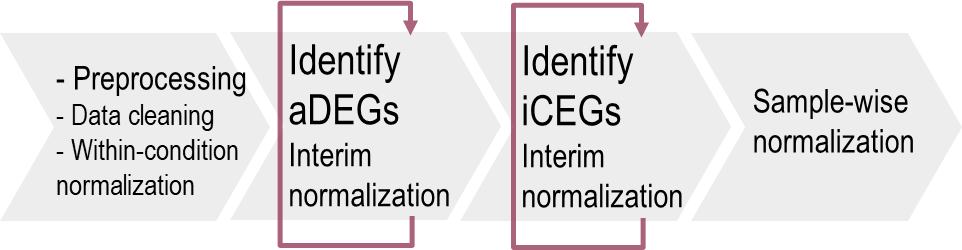

## Supplementary Tables

**Table S1**. Average of LFC MSE values associated with iCEGs restored by Cosbin and peer methods via different normalization strategies in mixture simulation study.


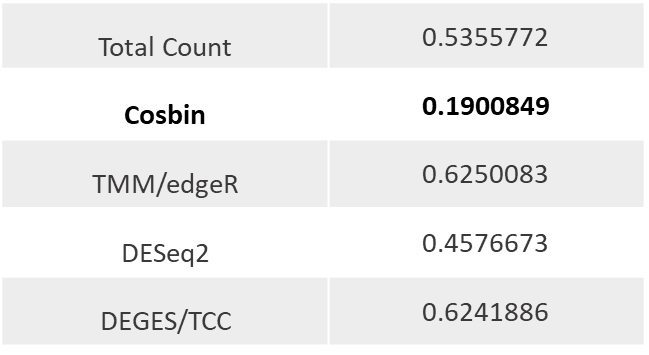


**Table S2**. (Top) Average dislocation of iCEGs (# iCEGs = 168) in degree and AUC of detecting general DEGs (# DEGs = 232) after normalization by Cosbin and four size-factor-based peer methods, summarized from TCC simulation studies. Note that the maximum possible iCEG dislocation in this case is about 55⁰. (Bottom) Average of LFC-MSE values associated with iCEGs restored by Cosbin and peer methods via different normalization strategies in TCC simulation study.


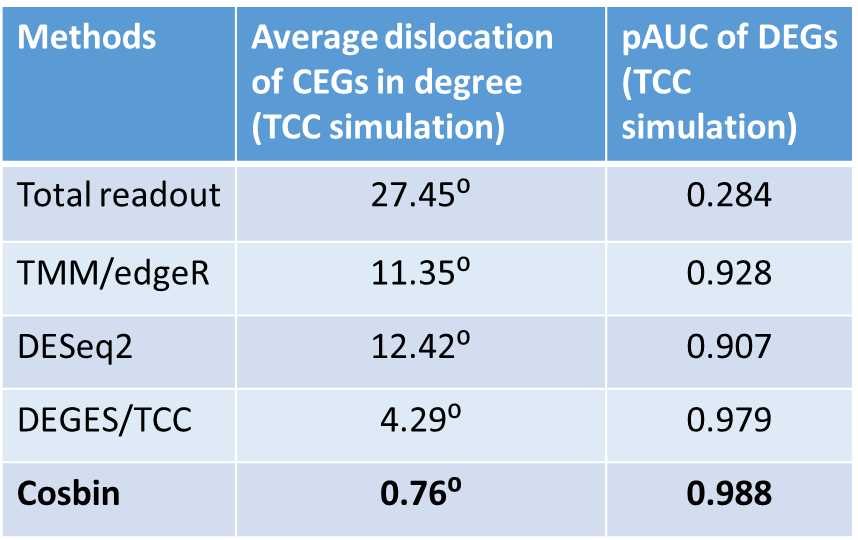


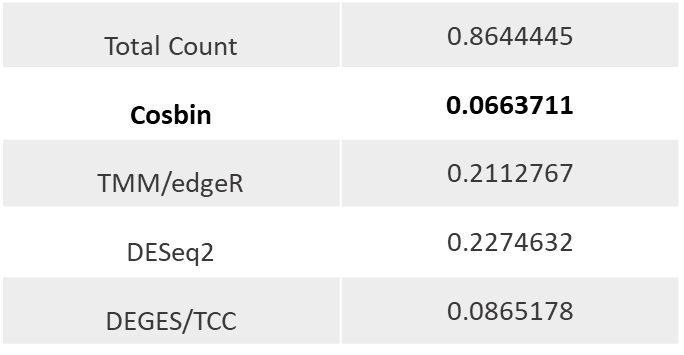


**Table S3.** List of top 72 SSG detected after Cosbin on proteomics data of pure samples.

| **Uniprot** | **Gene** | **MaxCos** | **Subtype** |
| --- | --- | --- | --- |
| P01023 | A2M | 0.996325 | FP |
| P35858 | IGFALS | 0.986678 | FP |
| P04114 | APOB | 0.980829 | FP |
| P05090 | APOD | 0.976805 | FP |
| P02649 | APOE | 0.983331 | FP |
| P04003 | C4BPA | 0.983988 | FP |
| P02671 | FGA | 0.98373 | FP |
| P02675 | FGB | 0.990368 | FP |
| P02679 | FGG | 0.991236 | FP |
| P00738 | HP | 0.993921 | FP |
| P01871 | IGHM | 0.981127 | FP |
| P01591 | JCHAIN | 0.99669 | FP |
| P19823 | ITIH2 | 0.980537 | FP |
| P55058 | PLTP | 0.993903 | FP |
| O00391 | QSOX1 | 0.977062 | FP |
| P35542 | SAA4 | 0.991518 | FP |
| P04004 | VTN | 0.980874 | FP |
| P01617 | IGKV2D-28 | 0.976271 | FP |
| P01621 | IGKV3-20 | 0.97075 | FP |
| P01625 | IGKV4-1 | 0.977167 | FP |
| P01714 | IGLV3-19 | 0.97709 | FP |
| P01764 | IGHV3-23 | 0.99168 | FP |
| P01767 | IGHV3-53 | 0.973074 | FP |
| P01834 | IGKC | 0.969471 | FP |
| P01861 | IGHG4 | 0.976097 | FP |
| P01876 | IGHA1 | 0.983901 | FP |
| P02647 | APOA1 | 0.983395 | FP |
| P02652 | APOA2 | 0.974894 | FP |
| P02656 | APOC3 | 0.978704 | FP |
| P04433 | IGKV3-11 | 0.989407 | FP |
| P10643 | C7 | 0.970063 | FP |
| O14818 | PSMA7 | 0.694827 | FS |
| O60701 | UGDH | 0.683484 | FS |
| O75131 | CPNE3 | 0.765425 | FS |
| O75369 | FLNB | 0.726902 | FS |
| O95373 | IPO7 | 0.699861 | FS |
| P16112 | ACAN | 0.68885 | FS |
| P17661 | DES | 0.681028 | FS |
| P17858 | PFKL | 0.717842 | FS |
| P20700 | LMNB1 | 0.749738 | FS |
| P20810 | CAST | 0.717193 | FS |
| P28289 | TMOD1 | 0.794207 | FS |
| P43121 | MCAM | 0.715572 | FS |
| P61457 | PCBD1 | 0.693923 | FS |
| P62879 | GNB2 | 0.785242 | FS |
| Q08211 | DHX9 | 0.706518 | FS |
| Q53TN4 | CYBRD1 | 0.736895 | FS |
| Q5T4S7 | UBR4 | 0.715596 | FS |
| Q6DD88 | ATL3 | 0.706456 | FS |
| Q7KZF4 | SND1 | 0.703849 | FS |
| Q86UP2 | KTN1 | 0.696156 | FS |
| Q92499 | DDX1 | 0.73185 | FS |
| Q92597 | NDRG1 | 0.742916 | FS |
| Q9BUF5 | TUBB6 | 0.706156 | FS |
| O00151 | PDLIM1 | 0.708398 | NL |
| O15061 | SYNM | 0.744163 | NL |
| P05787 | KRT8 | 0.842153 | NL |
| P17661 | DES | 0.687467 | NL |
| P24844 | MYL9 | 0.691911 | NL |
| P30837 | ALDH1B1 | 0.71162 | NL |
| P34932 | HSPA4 | 0.709182 | NL |
| P48059 | LIMS1 | 0.716689 | NL |
| P50552 | VASP | 0.703494 | NL |
| P55809 | OXCT1 | 0.704988 | NL |
| Q0ZGT2 | NEXN | 0.682592 | NL |
| Q13683 | ITGA7 | 0.705244 | NL |
| Q14515 | SPARCL1 | 0.73619 | NL |
| Q14BN4 | SLMAP | 0.746455 | NL |
| Q6UVK1 | CSPG4 | 0.712708 | NL |
| Q8IXB1 | DNAJC10 | 0.723565 | NL |
| Q9HCB6 | SPON1 | 0.829684 | NL |
| Q9Y4G6 | TLN2 | 0.695395 | NL |

**Table S4**. Average deviation degree of MGs restored by Cosbin, DEGES/TCC, Total Count, in benchmarking study.


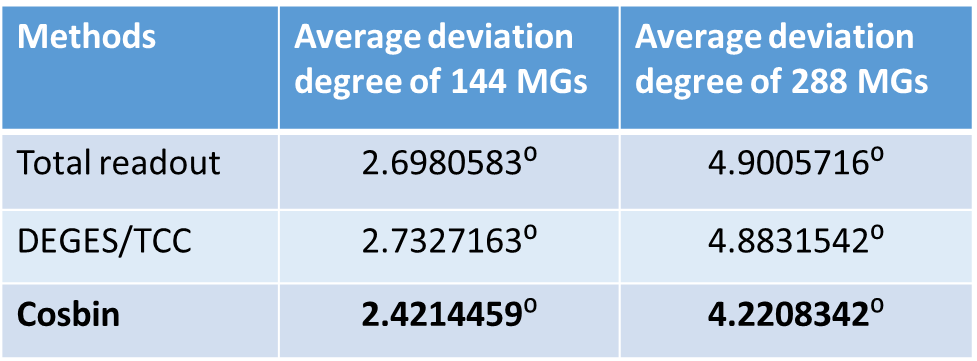


## Supplementary Figures


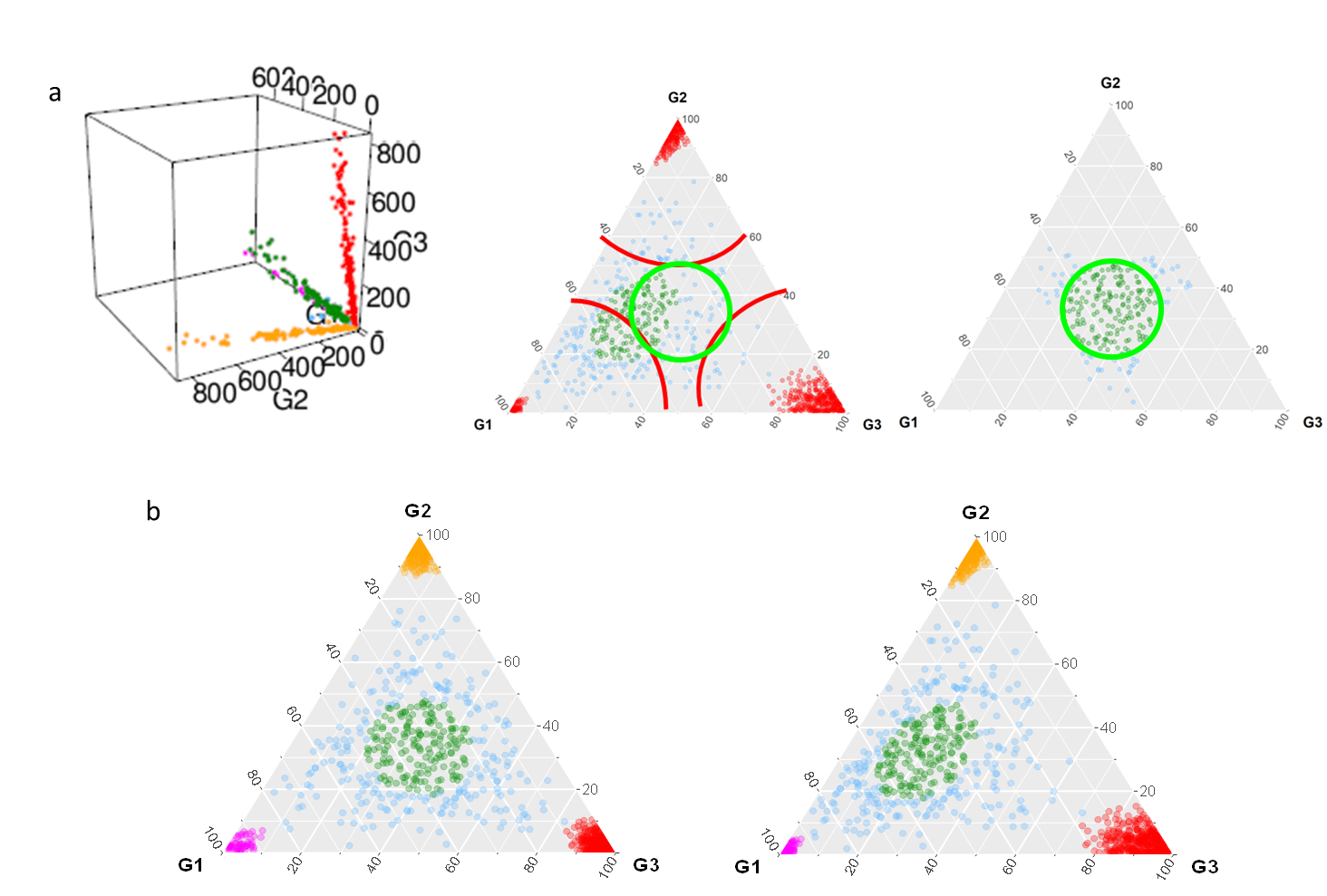


**Figure S1**. (a) An illustrative toy dataset and the scatter simplex, where significantly asymmetric DEGs, iCEGs, and remaining genes are color-coded. (b) Scatter simplex of the ground truth (left) and biased readout (right) in mixture simulations.


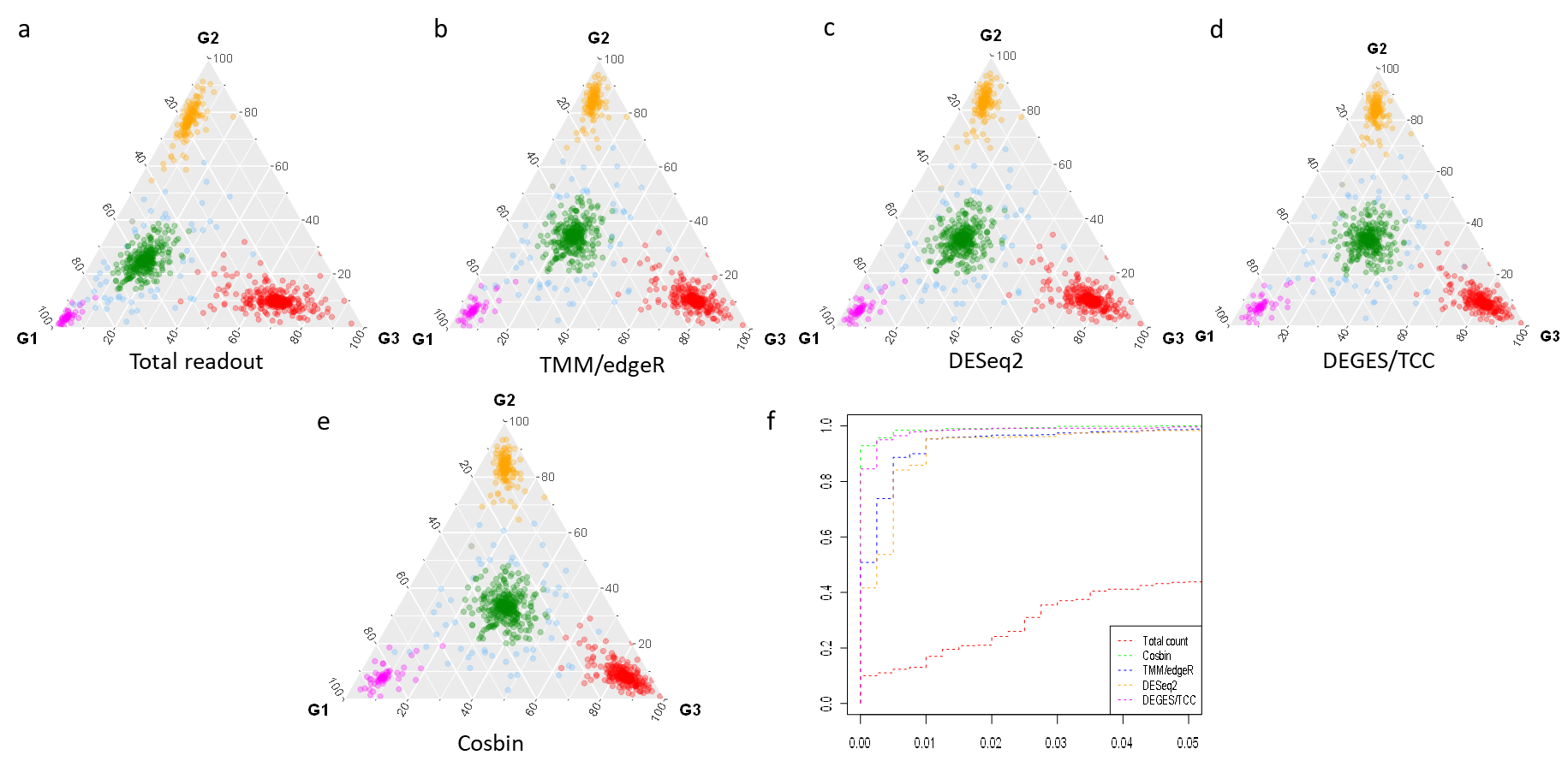


**Figure S2**. Comparative evaluation of Cosbin and four peer methods using TCC simulation data. (a-e) The location of iCEGs (green, # iCEGs = 168), significant aDEGs (red/orange/pink, # aDEGs = 600), and general DEGs (blue, # DEGs = 232), restored by Cosbin and four peer methods after normalization. (f) The pROC curves of detecting general DEGs against iCEGs by Cosbin and four peer methods.


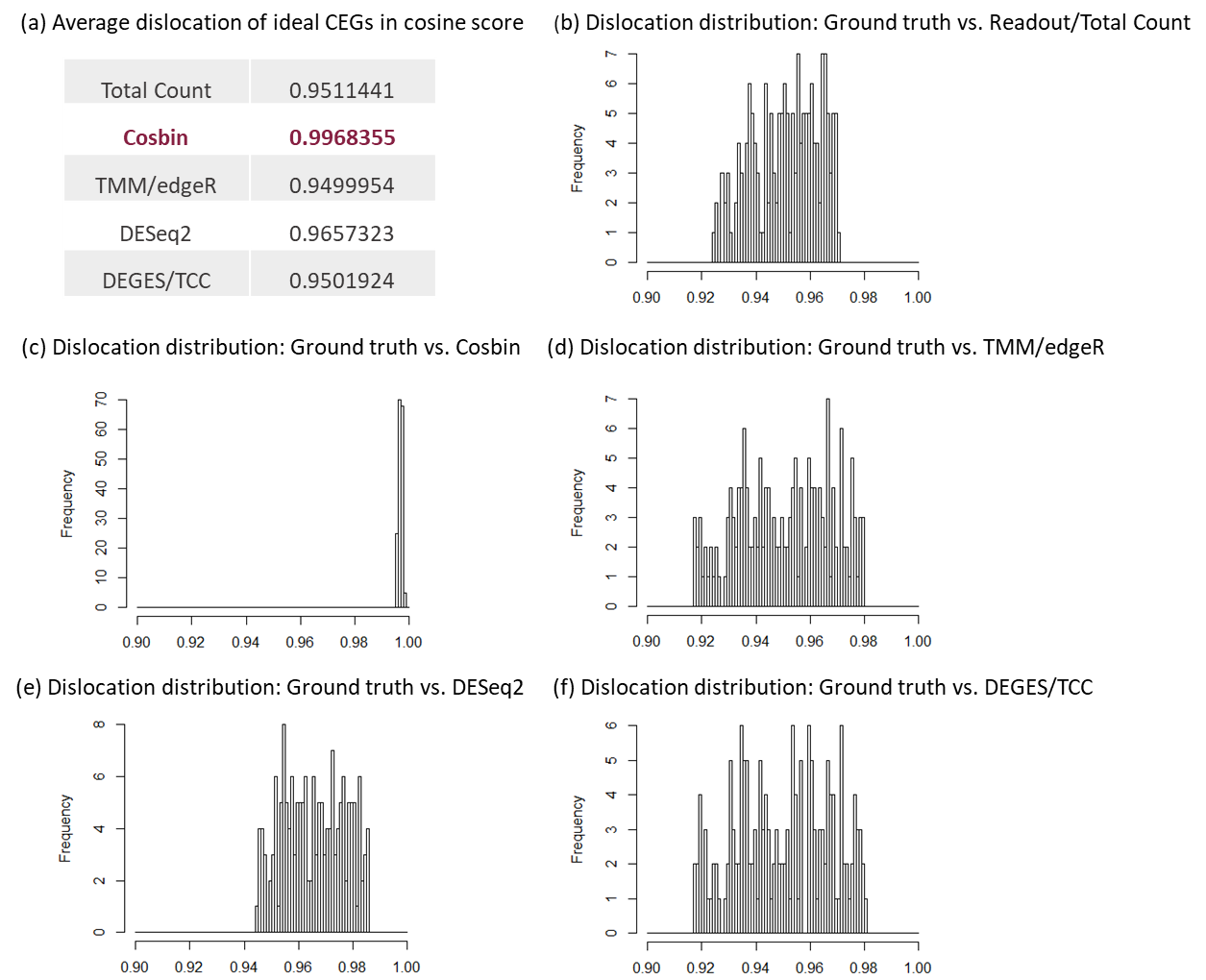


**Figure S3**. Summary statistics on the restored dislocation (in reference to ground truth location) of iCEGs by Cosbin and peer normalization methods in mixture simulation studies. (a) Average dislocation of iCEGs in cosine scores produced by various methods. (b-f) Restored-dislocation distributions of iCEGs associated with Total Count, Cosbin, TMM/edgeR, DESeq2, and DEGES/TCC, respectively.


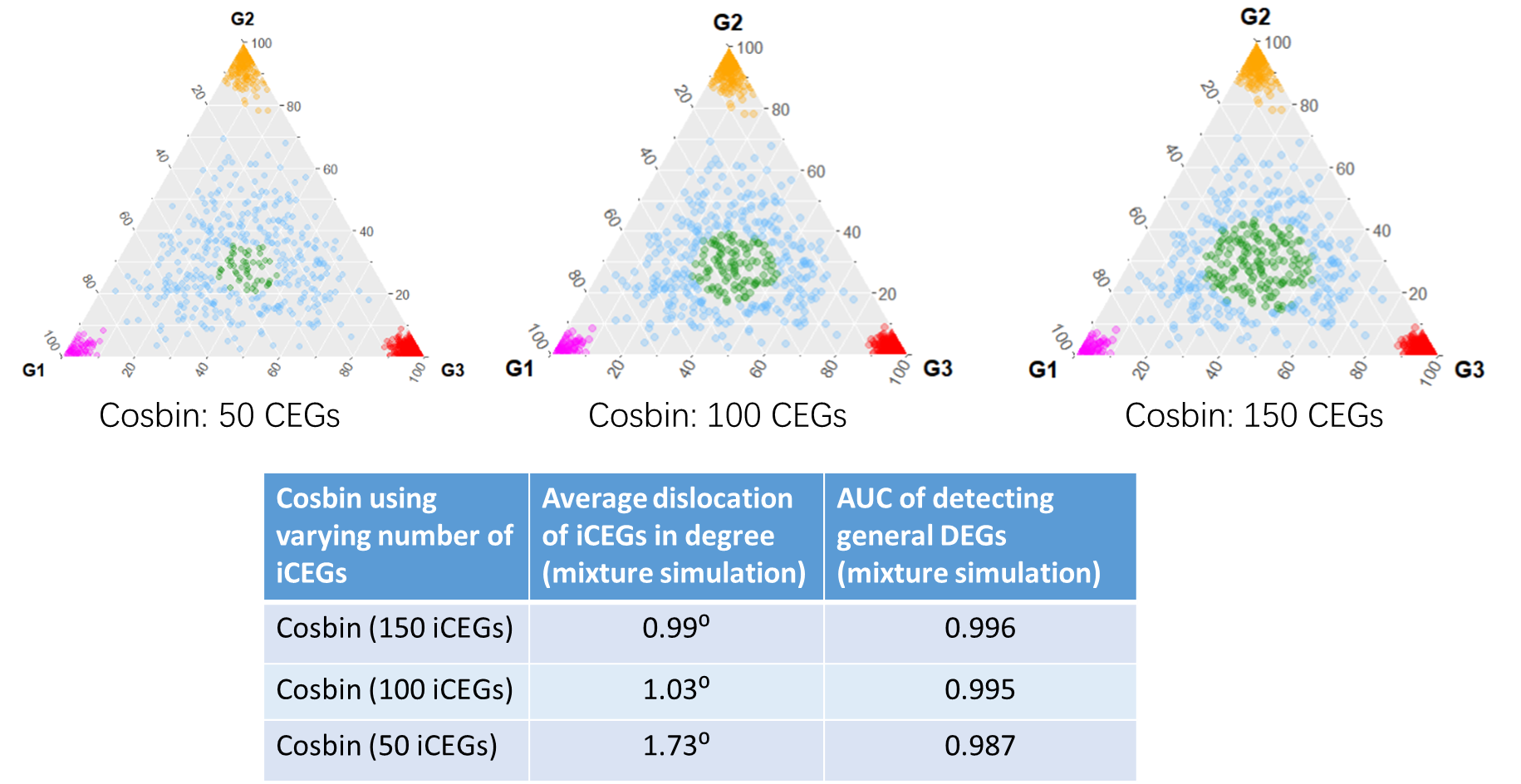


**Figure S4**. Evaluation on the robustness of Cosbin performance using varying number of iCEGs in the mixture simulation studies. (Top) The location of iCEGs (green, # iCEGs = 50, 100, 150 respectively), significant aDEGs (red/orange/pink, # aDEGs = 600), and general DEGs (blue, # DEGs = 350, 300, 250 respectively), restored by Cosbin after normalization. (Bottom) The average iCEG dislocations in degree, and AUC values of detecting general DEGs against iCEGs, after normalization-by-testing and size-factoring by Cosbin.


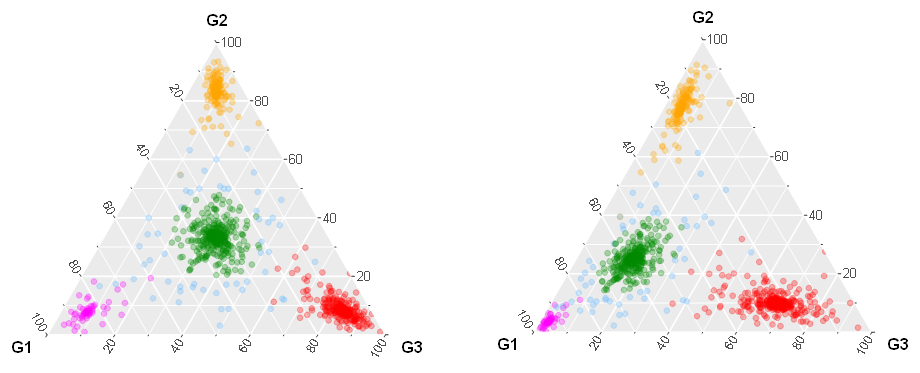


**Figure S5.** Scatter simplex of the ground truth (left) and biased readout (right) in TCC simulations.


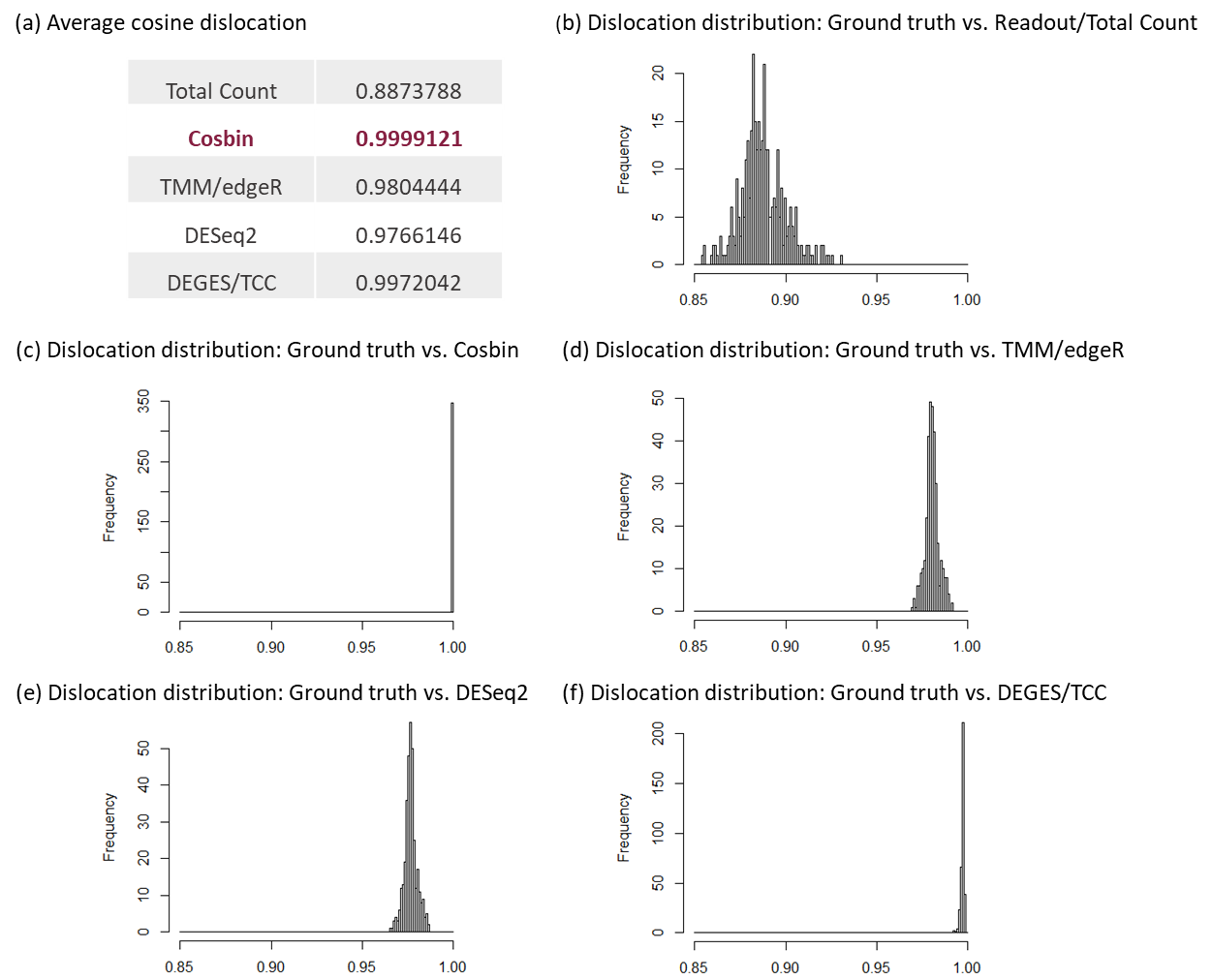


**Figure S6**. Summary statistics on the restored dislocation (in reference to ground truth location) of iCEGs by Cosbin and peer normalization methods in TCC simulation studies. (a) Average dislocation of iCEGs in cosine scores produced by various methods. (b-f) Restored-dislocation distributions of iCEGs associated with Total Count, Cosbin, TMM/edgeR, DESeq2, and DEGES/TCC, respectively.


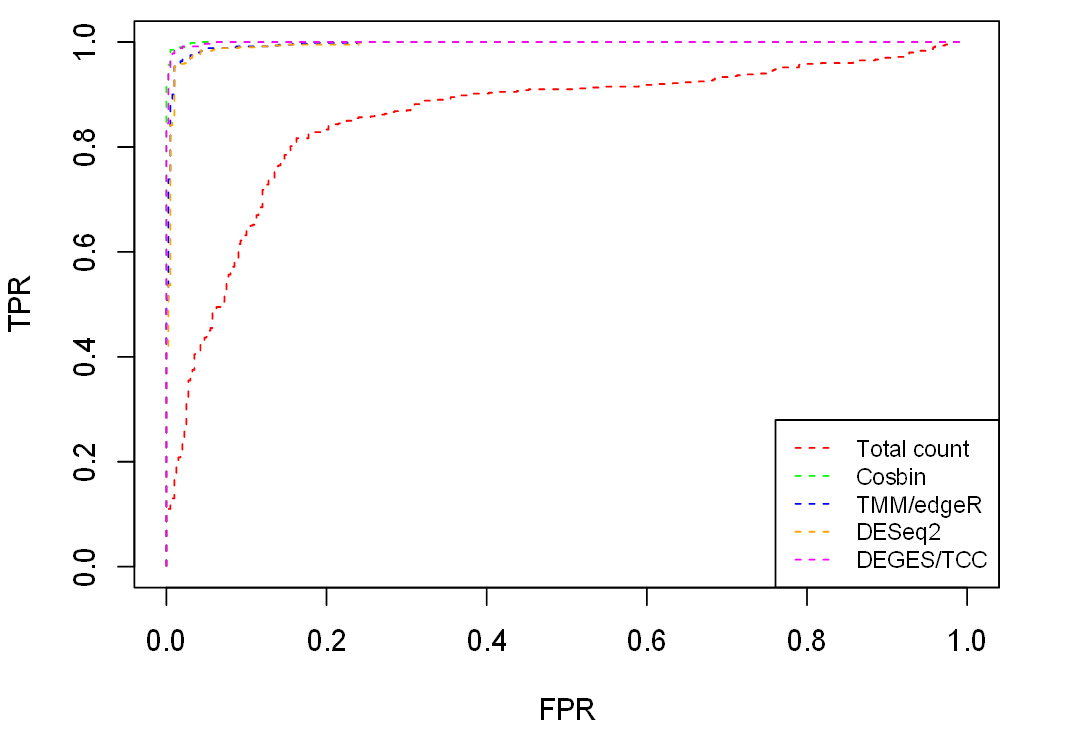


**Figure S7**. ROC curve of DEG (all non-CEGs subject to varying cosine score threshold) detection after normalization by Cosbin and peer methods in TCC simulation studies.


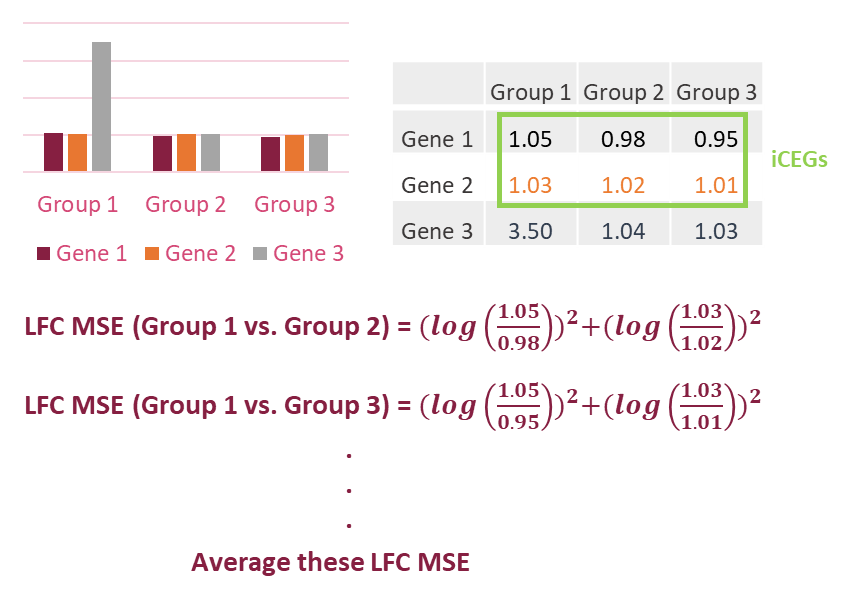


**Figure S8**. Illustrative description of LFC-MSE concept and calculation on iCEGs.


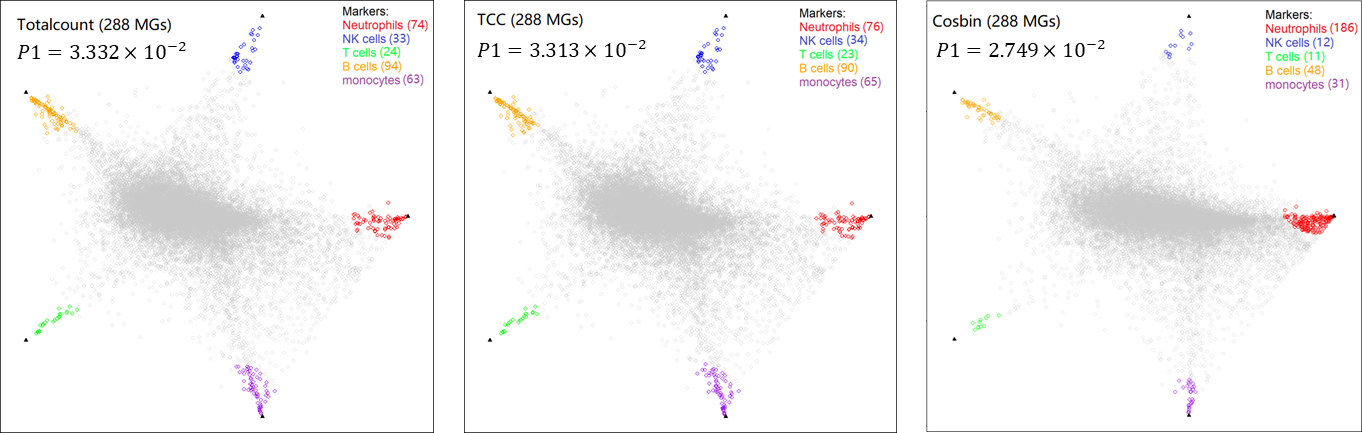


**Figure S9**. Scatter simplex of GSE28490 superimposed by the top 288 MGs (color-coded) detected after normalization by total count, TCC, and Cosbin, respectively; where black triangle indicates the ideal MG references.


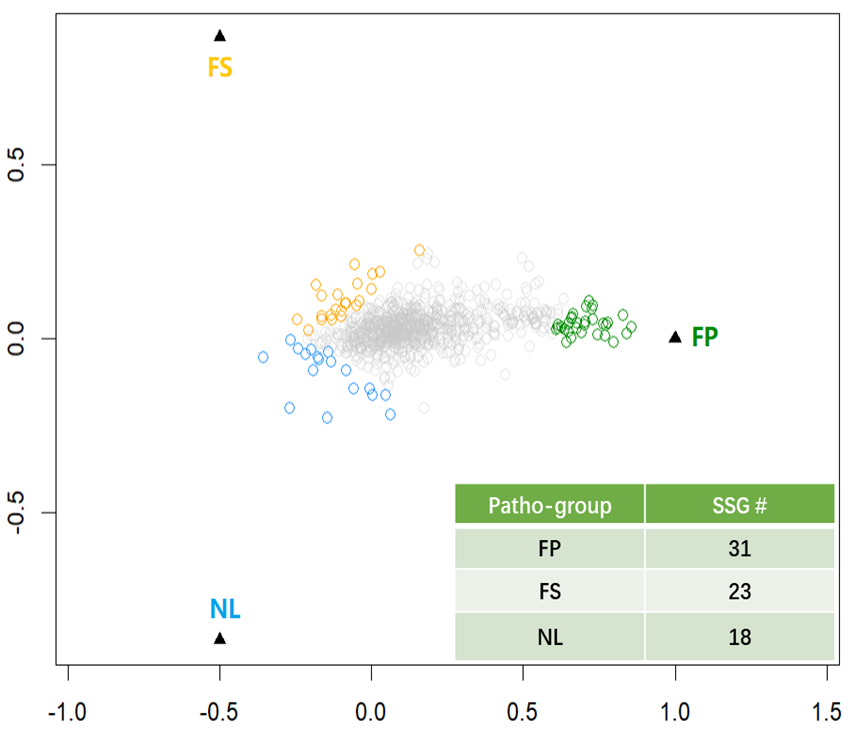


**Figure S10**. Scatter simplex of vascular proteomics data superimposed by the top 72 SSGs (color-coded) detected after normalization by Cosbin, where black triangle indicates the ideal MG references.


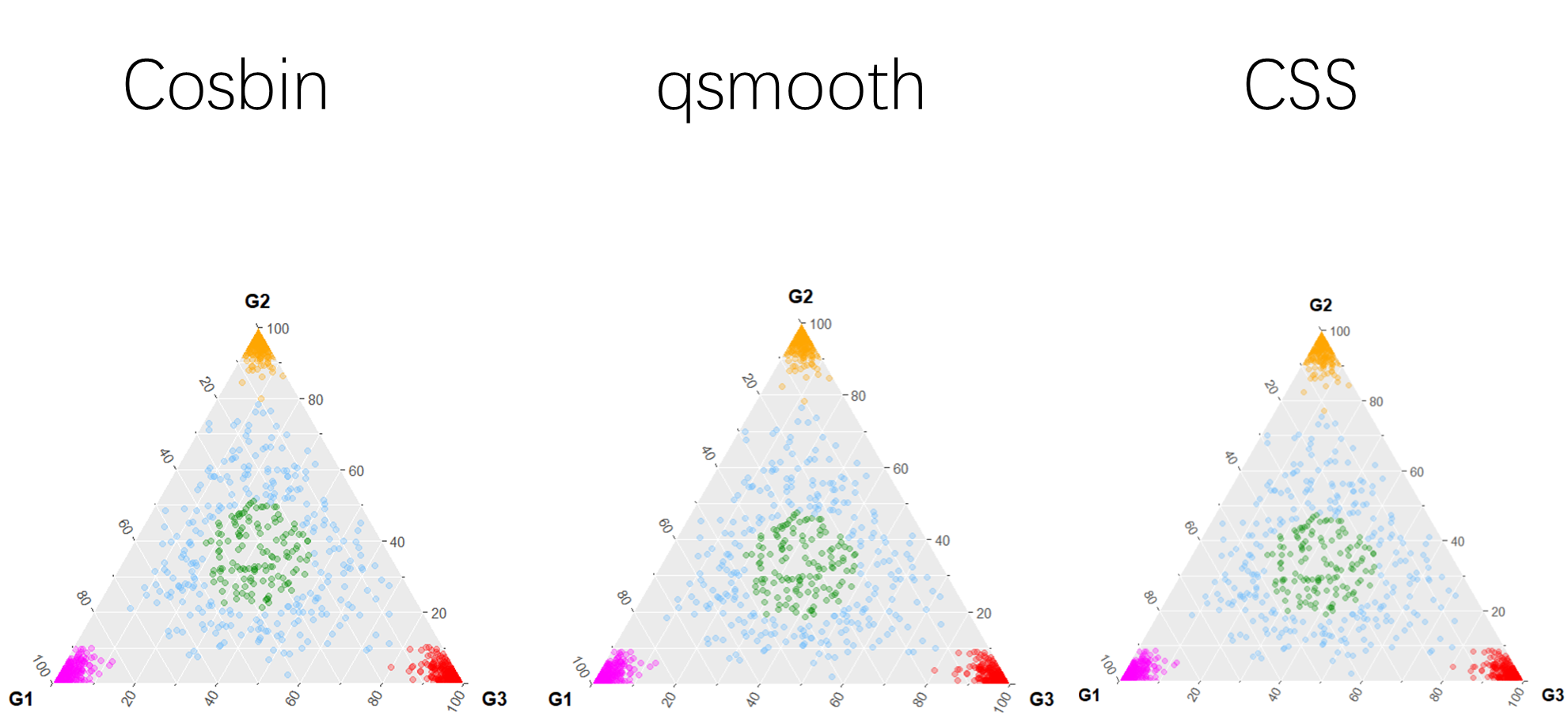


**Figure S11**. Experimental results of Cosbin, qsmooth and CSS when differential expressions are symmetric, showing that all methods perform equally good.

**
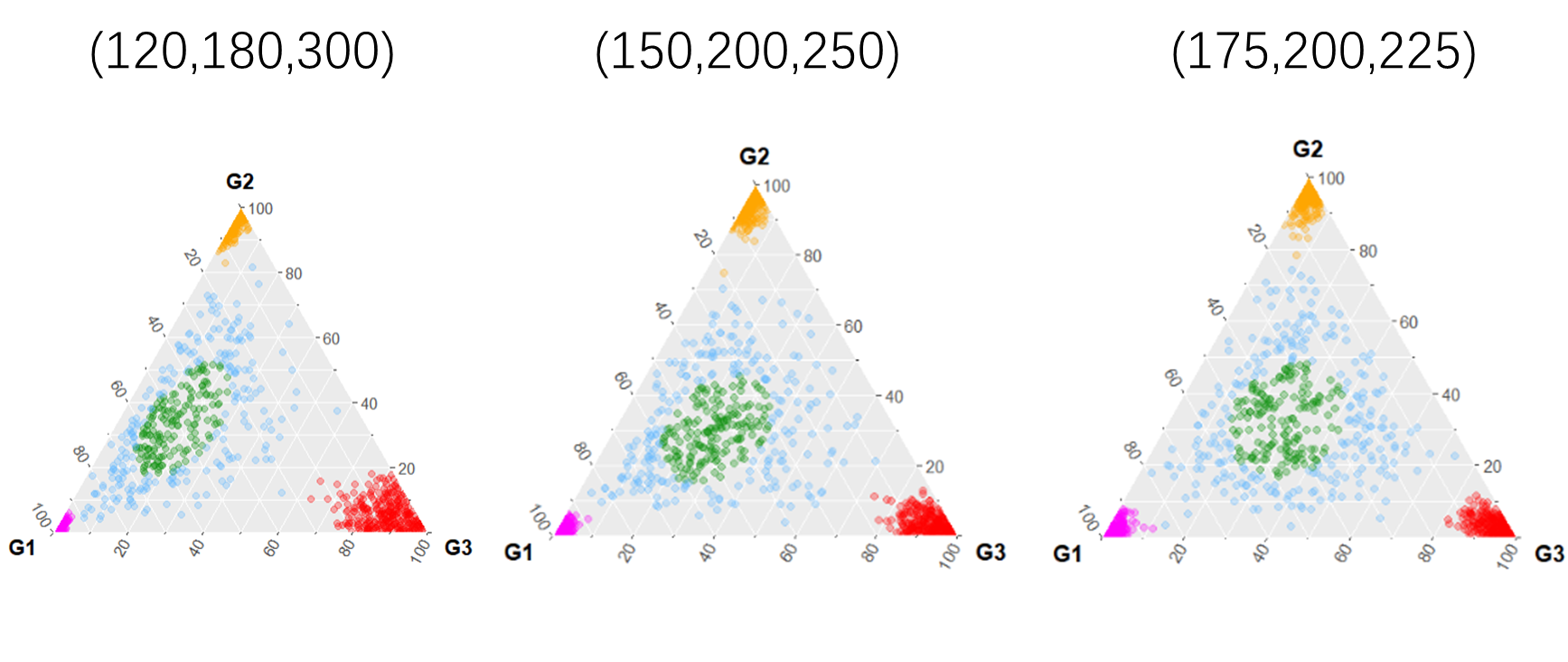
**

**Figure S12**. Experimental results to evaluate how the performance of global adjustment CSS method changes as the amounts of asymmetry in differential expression (with varying level of aDEG imbalance), showing that CSS performs better with less amounts of asymmetry in differential expression.

## References

Anders, S. and Huber, W. Differential expression analysis for sequence count data. *Nature Precedings* 2010:1-1.

Evans, C., Hardin, J. and Stoebel, D.M. Selecting between-sample RNA-Seq normalization methods from the perspective of their assumptions. *Brief Bioinform* 2018;19(5):776-792.

Hicks, S.C. and Irizarry, R.A. quantro: a data-driven approach to guide the choice of an appropriate normalization method. *Genome Biol* 2015;16:117.

Hicks, S.C.*, et al.* Smooth quantile normalization. *Biostatistics* 2018;19(2):185-198.

Hunt, G.J.*, et al.* dtangle: accurate and robust cell type deconvolution. *Bioinformatics* 2019;35(12):2093-2099.

Jo, J.*, et al.* Conventionally used reference genes are not outstanding for normalization of gene expression in human cancer research. *BMC Bioinformatics* 2019;20(Suppl 10):245.

Kadota, K., Nishiyama, T. and Shimizu, K. A normalization strategy for comparing tag count data. *Algorithms Mol Biol* 2012;7(1):5.

Kuhn, A.*, et al.* Cell population-specific expression analysis of human cerebellum. *BMC genomics* 2012;13:610.

Kuhn, A.*, et al.* Population-specific expression analysis (PSEA) reveals molecular changes in diseased brain. *Nat Methods* 2011;8(11):945-947.

Lu, Y.*, et al.* COT: an efficient and accurate method for detecting marker genes among many subtypes. *Bioinform Adv* 2022;2(1):vbac037.

Newman, A.M.*, et al.* Robust enumeration of cell subsets from tissue expression profiles. *Nat Methods* 2015;12(5):453-457.

Newman, A.M.*, et al.* Determining cell type abundance and expression from bulk tissues with digital cytometry. *Nat Biotechnol* 2019;37(7):773-782.

Paulson, J.N.*, et al.* Differential abundance analysis for microbial marker-gene surveys. *Nat Methods* 2013;10(12):1200-1202.

Robinson, M.D. and Oshlack, A. A scaling normalization method for differential expression analysis of RNA-seq data. *Genome biology* 2010;11(3):1-9.

Sun, J.*, et al.* TCC: an R package for comparing tag count data with robust normalization strategies. *BMC bioinformatics* 2013;14(1):1-14.

Sun, J.*, et al.* TCC: an R package for comparing tag count data with robust normalization strategies. *BMC Bioinformatics* 2013;14:219.

Wang, J., Roeder, K. and Devlin, B. Bayesian estimation of cell type-specific gene expression with prior derived from single-cell data. *Genome Res* 2021.

Zhao, S., Ye, Z. and Stanton, R. Misuse of RPKM or TPM normalization when comparing across samples and sequencing protocols. *RNA* 2020;26(8):903-909.

Zhong, Y.*, et al.* Digital sorting of complex tissues for cell type-specific gene expression profiles. *BMC Bioinformatics* 2013;14:89.
